# In vivo imaging of free radicals produced by multivitamin-mineral supplements

**DOI:** 10.1186/s40795-015-0025-7

**Published:** 2015-11-14

**Authors:** Alexander B. Rabovsky, Garry R. Buettner, Bruno Fink

**Affiliations:** 1 Research & Development, Melaleuca Inc, 4609 West 65th South, Idaho Falls, ID 83402, USA; 2 Free Radical and Radiation Biology, The University of Iowa, Iowa City, IA, USA; 3 Noxygen Science Transfer & Diagnostics GmbH, Elzach, Germany

**Keywords:** Vitamins, Minerals, Electron paramagnetic resonance, Free radical, Ascorbate, Imaging, Oxidation

## Abstract

**Background:**

Redox active minerals in dietary supplements can catalyze unwanted and potentially harmful oxidations.

**Methods:**

To determine if this occurs in vivo we employed electron paramagnetic (EPR) imaging. We used 1-hydroxy-3-carboxy-2,2,5,5-tetramethylpyrrolidine (CPH) as a reporter for one-electron oxidations, *e.g*. free radical-mediated oxidations; the one-electron oxidation product of CPH, 3-carboxy-2,2,5,5-tetramethyl-1-pyrrolidinyloxy (CP^•^), is a nitroxide free radical that is relatively persistent in vivo and detectable by EPR. As model systems, we used research formulations of vitamin mineral supplements (RVM) that are typical of commercial products.

**Results:**

In in vitro experiments, upon suspension of RVM in aqueous solution, we observed: (1) the uptake of oxygen in the solution, consistent with oxidation of the components in the RVM; (2) the ascorbate free radical, a real-time indicator of ongoing oxidations; and (3) when amino acid/oligosaccharide (AAOS; glycinate or aspartate with non-digestible oligofructose) served as the matrix in the RVM, the rate of oxidation was significantly slowed. In a murine model, EPR imaging showed that the ingestion of RVM along with CPH results in the one-electron oxidation of CPH by RVM in the digestive system. The resulting CP^•^ distributes throughout the body. Inclusion of AAOS in the RVM formulation diminished the oxidation of CPH to CP^•^ in vivo.

**Conclusions:**

These data demonstrate that typical formulations of multivitamin/multimineral dietary supplements can initiate the oxidation of bystander substances and that AAOS-complexes of essential redox active metals, *e.g*. copper and iron, have reduced ability to catalyze free radical formation and associated detrimental oxidations when a part of a multivitamin/multimineral formulation.

## Background

Although nutritional supplements are not intended to substitute for a healthy, varied diet, millions of people complement their daily food intake with dietary supplements to ensure adequate intake of essential nutrients required for optimal health. Formulations of multivitamin supplements typically include oxidation-sensitive vitamins, such as vitamins C and E, as well as redox active minerals, such as iron and copper. These redox active transition metals can serve as catalysts for the oxidation of organic compounds. For example, adventitious, trace levels of iron and copper in near-neutral phosphate buffer readily catalyze the oxidation of ascorbate [1–3]. Ferric iron is a standard reagent used to oxidize tocopherols to their corresponding quinones [4], which are inactive as antioxidants; in fact quinones can function as pro-oxidants [5, 6]. The combination of these minerals and ascorbate in dietary supplements can catalyze unwanted and potentially harmful oxidations [3, 7–10]. For example, the metal-catalyzed oxidation of ascorbate can lead to the oxidation of substances and structures in cells and tissues [3, 11]. The combination of iron and ascorbate, referred to as the Udenfriend system, is a standard approach to oxidize organic substances [12]. Thus, these metals could bring about the loss of antioxidants as well as initiate potentially harmful oxidation reactions in cells and tissues before absorption by the digestive system.

Because we had previously observed that dietary supplements can catalyze oxidations in vitro [10], we hypothesized that similar oxidations could also occur in vivo. Here we prepared research multivitamin/multi mineral formulations (RVM) based on RDA guidelines with minerals as typical inorganic complexes or with amino acid/oligosaccharide (AAOS; glycinate or aspartate with non-digestible oligofructose) serving as the coordinating ligands and matrix for the minerals in the RVM. The abilities of these formulations to initiate oxidation processes in vitro and in vivo were examined. As reporters on these oxidations, we used oxygen uptake, ascorbate radical formation, and the oxidation of CPH to CP^•^, which can be monitored by EPR both in vitro and in vivo. The oxidation of CPH to CP^•^ reports on one-electron (free radical) oxidations. We used EPR imaging to determine if formulations of multivitamin/multimineral supplements can initiate free radical oxidations in vivo.

## Methods

### Materials

Oxygen-Sensitive Label (OSL; tetrathiatriarylmethyl radical), CPH (1-hydroxy-3-carboxy-2,2,5,5-tetramethylpyrrolidine), CP^•^ (3-carboxy-2,2,5,5-tetramethyl-1-pyrrolidi ne-1-oxyl, CAS# 50525-83-2 and 2154-68-9), CAT1-H (1-hydroxy-2,2,6,6-tetramethylpiperidin-4-yl-trimethylammonium chloride•HCl), DFO (deferoxamine, methanesulfonate salt, CAS# 138-14-7), DETC (diethyldithiocarbamic acid, sodium salt trihydrate CAS# 20624-25-3), and Teflon^®^ microtubes (50 µL) were from Noxygen Science Transfer & Diagnostics, GmbH, Elzach, Germany. The amino acid/oligosaccharide (AAOS; glycinate or aspartate with non-digestible oligofruictose) serving as the matrix for the minerals were prepared as previously described [10].

### RVM formulation

Two formulations of the research vitamins and minerals (RVM) were prepared with identical content of vitamins and excipients; the two formulations differed only by the sources of mineral: AAOS or inorganic (sulfates, chlorides, and oxides), Table 1. Vitamin A, vitamin C, vitamin D, vitamin E, vitamin K, thiamin, riboflavin, niacin, vitamin B6, folate, vitamin B12, biotin, pantothenic acid, microcrystalline cellulose, croscaramellose sodium, silicone dioxide, magnesium stearate, and carnauba wax were purchased from Sigma Chemical, Co., St. Louis, MO. Calcium carbonate, magnesium oxide, potassium iodide, copper sulfate, copper gluconate, iron sulfate, zinc sulfate, manganese sulfate, chromium chloride, and sodium molybdate were purchased from Spectrum Chemicals and Laboratory Products, New Brunswick, NJ.

The research multivitamin/multi mineral formulations were prepared based on RDA guidelines (Daily Values for Nutrition Labeling, 21 CFR §101.9(c) and CFR §101.36(b)(2)(ii)(B)) [13]. In addition to the active ingredients, typical excipients used in the tablet pressing and coating process included: microcrystalline cellulose, croscaramellose sodium, magnesium stearate, silicon dioxide, coating cellulose, and carnauba wax.

Supplement manufacturers often split multivitamins and multi minerals into two supplements, thereby allowing them to include all ingredients at a level of 100 % Daily Value (DV). If this is not the case, they will commonly decrease the level of select minerals to avoid tablets or capsules that are too large for consumer comfort. Here, the redox inactive minerals, magnesium and calcium, are only at 10 % DV in the RVMs formulated for this study.

### Kinetics of oxygen consumption and ascorbate oxidation

Formulation powders (150 mg) were suspended in 20 mL of 100 mM HCl. The suspension was then mixed for 5 min at room temperature with the pH being maintained at 2.5 ± 0.2 by addition of HCl. Aliquots were then mixed with Oxygen-Sensitive Label and subsequently diluted with carbonate buffer (50 mM, pH 7.2). The final concentrations of copper and iron were 26 and 270 µM, respectively; the final concentration of OSL was 4 µM. The suspension was transferred to a 50 µL glass micropipette (Hirschmann Laborgeräte GmbH & Co. KG, Eberstadt, Germany); ascorbate radical and oxygen consumption were monitored simultaneously. The initial concentration of oxygen in the air-saturated aqueous solutions of these experiments was taken as 210 µM (altitude of 1445 m) [14].

A Bruker E-SCAN EPR spectrometer with a Temperature and Gas Controller (BIO III, Noxygen Science Transfer & Diagnostics, GmbH, Elzach, Germany) was used to monitor oxygen consumption and the changes in the concentration of ascorbate free radical. EPR spectrometer settings were: center field *g* = 2.01; microwave power, 20 mW; modulation amplitude, 0.98 G; sweep rate, 10 G/5.24 s; number of scans, 3; for 1024-point spectrum.

### CPH oxidation to CP^•^ in vitro

Stock solutions of CPH were prepared in Krebs HEPES buffer and stored at −80 °C. CPH working solution was prepared in carbonate buffer (50 mM, pH 7.2) containing CPH (200 µM), DFO (25 µM), and DETC (5 µM)–final concentrations quoted. Powders were prepared as described in “Kinetics of oxygen consumption and ascorbate oxidation”. Suspensions were transferred to a 50 µL Teflon microtube. Spectra were collected with a Bruker E-SCAN spectrometer equipped with a Temperature & Gas Controller (Noxygen GmbH, Germany). EPR instrument settings were: center field, *g* = 2.01; microwave power, 20 mW; modulation amplitude, 2.2 G; sweep rate, 80 G/5.24 s; number of scans, 10; a 1024-point spectrum; total experimental time, ≈28 min. Temperature and Gas Controller parameters were: temperature, 37 °C; pressure, 25 mmHg; oxygen, 7.4 %; and carbon dioxide, 0.5 %.

### Free radical formation in vivo

C57BL/6J mice (7- to 8-week old, 8 groups, 5 per group) were used to examine free radical formation in vivo by the different RVM formulations. Animals were handled in accordance with the Animal Welfare Act (AWA) (7 U.S.C. § 2131) and the German Animal Welfare Act (Tierschutzgesetz); all protocols were approved by the regional commission Emmendingen for animal care under registration number DE08316100121 accordingly § 3 of regulation #1069/2009.

RVM formulations were powdered and then suspended in 0.9 % NaCl solution (pH ≤ 3) containing CPH at a final concentration of 1 mM; *e.g*. 3.57 mg of RVM/200 µL for a 25 g mouse, the exact amount of RVM was adjusted for the actual weight of the mouse. This amount corresponds to approximately 10 times the recommended dose for humans. The oxygen tension in this mixture was adjusted to 40 mmHg of oxygen, typical of the concentration of oxygen in the digestive system [unpublished data obtained in the Noxygen Science Transfer & Diagnostics GmbH laboratories]. The RVM suspension (200 µL) was immediately administered by oral gavage. Fifteen min after administration of the RVM/CPH mixture, mice were sacrificed without pain in compliance with Tierschutzgesetz guidelines for harvesting samples of gastric, intestine, and bladder fluids as well as samples of venous blood taken from the right heart ventricle. Equal volumes (30 µL) of collected samples were analyzed using a BenchTop EPR spectrometer E-Scan (Noxygen Science Transfer & Diagnostics GmbH, Germany) for the level of CP-radical (CP^•^). The EPR instrument settings were: center field, *g* = 2.01; field sweep, 60 G; microwave power, 20 mW; magnetic field modulation, 100 MHz; modulation amplitude, 2.0 G; conversion time, 80.24 ms; detector time constant, 20.96 ms; and sweep time, 60 s.

To verify the distribution of CP^•^ into the blood stream as well as determine the nonspecific oxidation of CPH we administered to two groups of mice by oral gavage solutions of 1.0 mM of CP^•^ or CPH (200 µL per 20 g BW) containing no RVM.

### EPR imaging of free radical formation in vivo

C57BL/6J mice (7- to 8-week old) were used to image in vivo free radical formation, *i.e*. formation of CP^•^, by the different RVM formulations (amounts and concentrations were the same as described above). Five and 20 min after administration of the RVM/CPH mixture the digestive system was imaged using an L-Band EPR-Spectrometer ELEXSYS E540 (Bruker Biospin GmbH, Germany). Before and during data acquisition mice were anesthetized using 2.2 % isoflurane. Mice were positioned in a 36 mm small-animal cavity equipped with an automatic matching control system. EPR instrument settings were: center field, *g* = 2.01; field sweep, 60 G; microwave power, 40 mW; magnetic field modulation, 1 GHz; modulation amplitude, 3.0 G; conversion time, 20.24 ms; detector time constant, 40.96 ms; image field of view, 25 mm; acquired angles, 31; gradient, 24 G/cm. The 2D EPR image was constructed employing the following strategies: zerothorder baseline correction; FT deconvolution using a gaussian window with a width of 0.15 mm; and filtered back projection.

## Results and discussion

### RVM formulation

It is not possible to compare directly the potential oxidative chemistry of different multivitamins/minerals supplements that are on the market due to interferences from the different matrices of the formulations. To overcome this barrier, research formulations (RVM) were prepared with identical matrices and vitamin content, differing only in the forms of minerals included in the formulations, Table 1. Using these formulations we examined the oxidations initiated by these formulations in vitro and in vivo.

### Oxygen uptake by RVM formulations

**Fig. 1:**
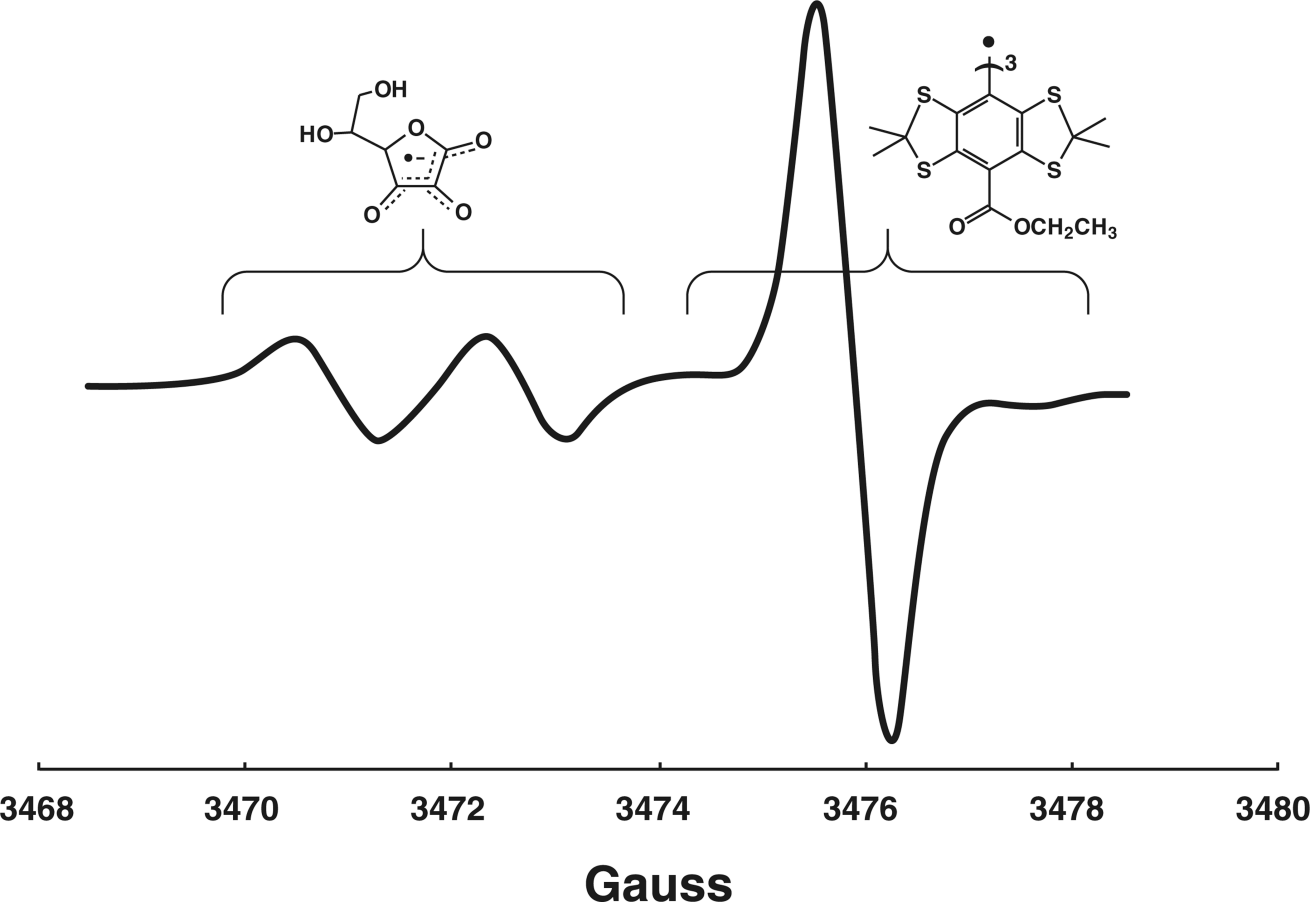
The ascorbate free radical and OSL can be monitored simultaneously by EPR. The spectra of ascorbate free radical (*g* = 2.005, a^H^ = 1.8 G) and the oxygen sensitive label (tetrathiatriarylmethyl radical; *g* = 2.003) have no significant overlap and their concentrations are low enough so that each can be analyzed independently without concern of interactions

**Fig. 2:**
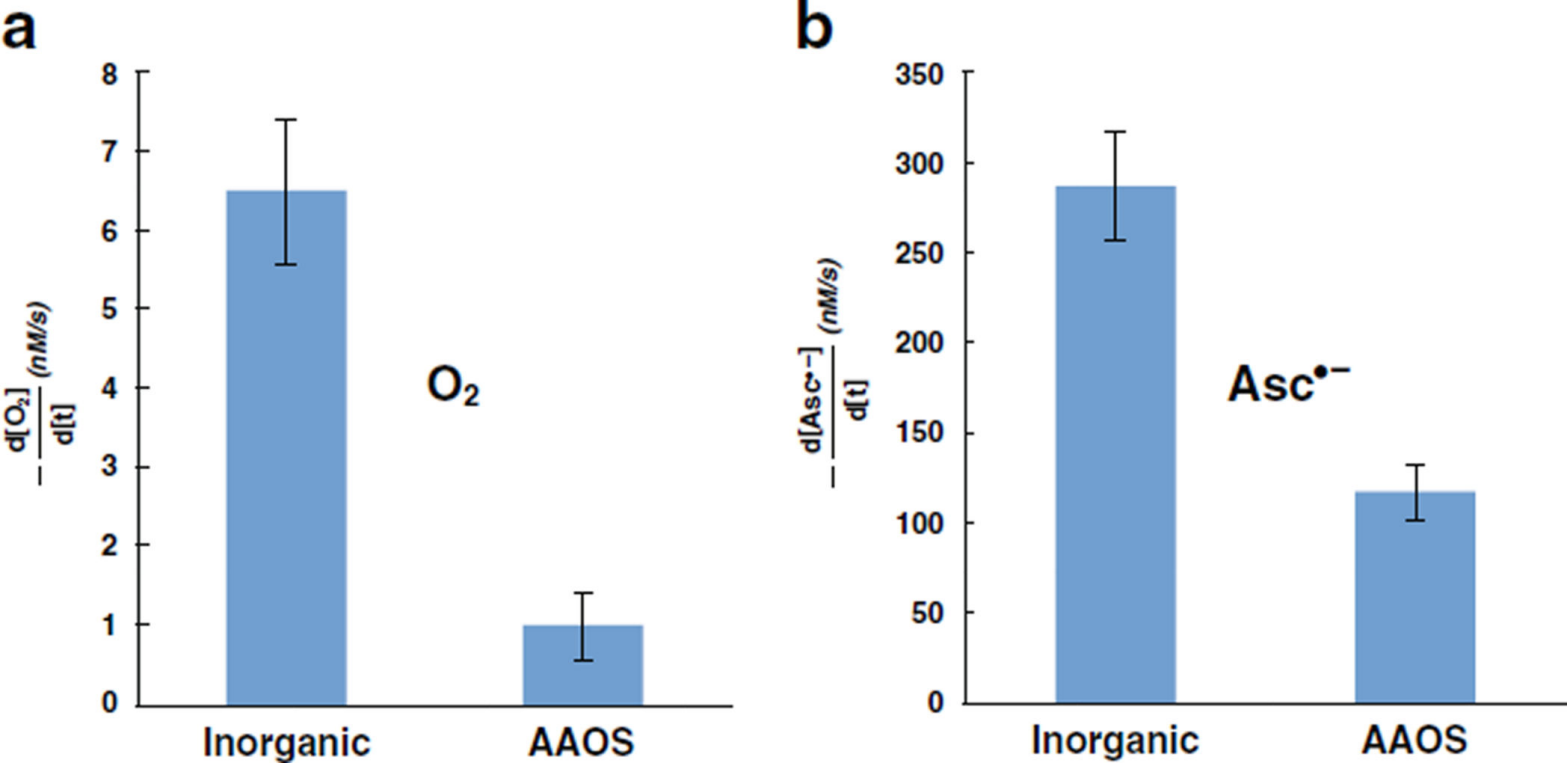
The rate of oxidation of RVM was greater when formulated with inorganic components compared to AAOS formulations. **a** rate of oxygen consumption of the two different formulations of RVM; **b** rate of loss of ascorbate radical. These results represent the mean and standard error of *3* experiments where loss of oxygen and ascorbate radical were monitored simultaneously by EPR in the samples

Because the uptake of oxygen is at the foundation of oxidation processes we monitored the rate of loss of oxygen in aqueous suspensions of RVM formulations. The concentration of oxygen in aqueous solution can be monitored by several approaches, including EPR [15]. Because the OSL did not react with the materials in the RVM suspensions, we used the OSL EPR signal amplitude as a measure of the oxygen concentration in solution, Fig. 1 [16–18]. The rates of oxygen uptake by the RVM formulations in near-neutral solutions were approximately linear until the concentration reached approximately 100 µM. As seen in Fig. 2a, AAOS decreased the rate of oxidation by more than 50 % compared to the inorganic formulation of RVM (d[O_2_]/dt = −280 nM s^−1^ for the inorganic RVM vs. only −120 nM s^−1^ for the AAOS RVM.

### RVM results in formation and rapid loss of Asc•-

The ascorbate radical and OSL can be monitored simultaneously by EPR, Fig. 1. Because the concentration of each radical is low (4 µM or less) and there is no significant spectral overlap, spectral analysis can be made without concern for interactions. RVM solutions showed the presence of the ascorbate free radical, a marker for the ongoing oxidation of ascorbate [19]. If the radical flux is low compared to ascorbate (*i.e*. the oxidizing free radical is the “limiting reagent”), then the ascorbate radical is an excellent measure of the ongoing flux of oxidants in the system; however, if the ascorbate is a limiting reagent, then it will be rapidly consumed with an associated decrease in the ascorbate radical over time. We observed that both RVM formulations resulted in a time-dependent loss of the EPR signal of the ascorbate free radical. However, the rate of loss of ascorbate radical with the inorganic formulation of RVM was more than six times greater than with the AAOS formulation (−6.5 nM s^−1^ vs. −1.0 nM s^−1^), Fig. 2b.

Informative is that the shapes of the curves for oxygen consumption and loss of ascorbate radical had parallels; initially each was approximately linear, then each had a clear slowing and loss of linearity at similar times (when approximately 100 µM of dioxygen had been consumed). The initial concentration of ascorbate in the solution being monitored by EPR was 60 µM while that of O_2_ is 210 µM. The oxidation of ascorbate to dehydroascorbic acid is a two-electron oxidation; this would result in the stoichiometric loss of 60 µM of O_2_ if H_2_O_2_ is the final reduction product or 30 µM of O_2_ if H_2_O is the final reduction product. The loss of more than 60 µM indicates that not only ascorbate is being oxidized, but other components of the RVM. Taken together, both oxygen consumption and the loss of ascorbate radical are consistent with RVM initiating oxidation processes in an aqueous environment. The inorganic formulation of RVM has a much greater rate of oxidation, demonstrating the importance of the form of the redox active metals in these oxidations.

### RVM oxidizes CPH by one-electron; CPH as a tool

In another approach to determine if the RVM formulations can initiate one-electron oxidations we used the oxidation of a hydroxylamine to its corresponding nitroxide [20–24]. CPH is a five-membered ring hydroxylamine that can undergo a one-electron oxidation to its corresponding nitroxide, CP^•^ (Reaction 1), which can be readily observed by EPR. We chose a five-membered hydroxylamine because its corresponding nitroxide is more resistant to reduction than six-membered ring nitroxides [20]; thus, once the five-membered ring nitroxide is formed it would be more persistent than a six-membered ring nitroxide in solutions that contain reducing agents, such as ascorbate, as well as in vivo. In addition, at near-neutral pH the carboxyl group will be unprotonated, which will influence the distribution of the nitroxide in in vivo EPR imaging experiments.


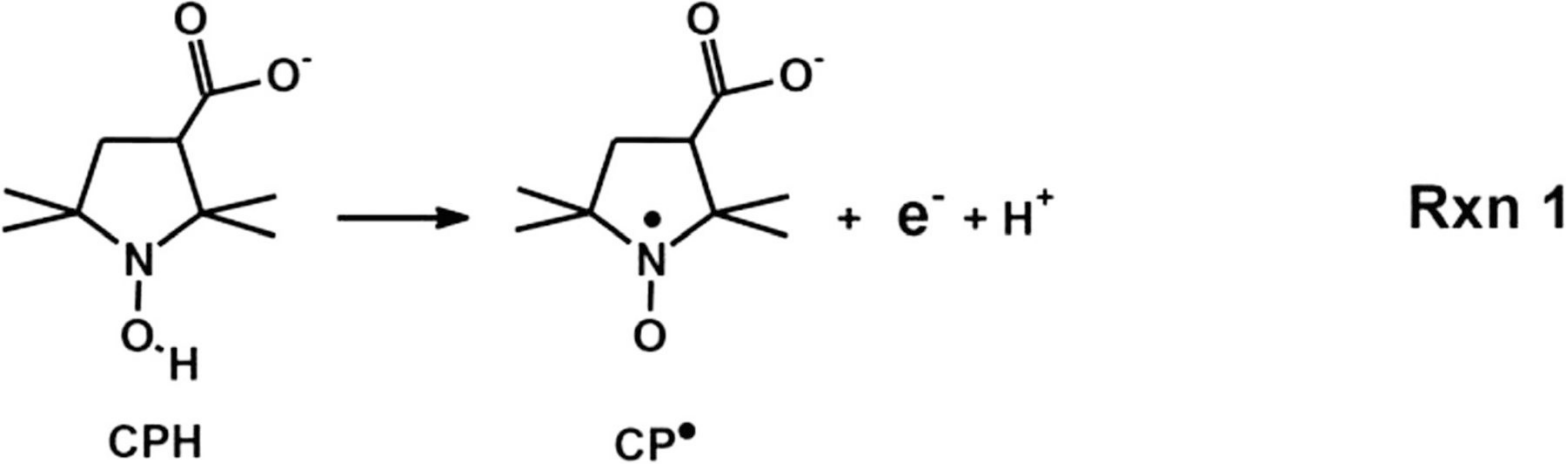


**Fig. 3:**
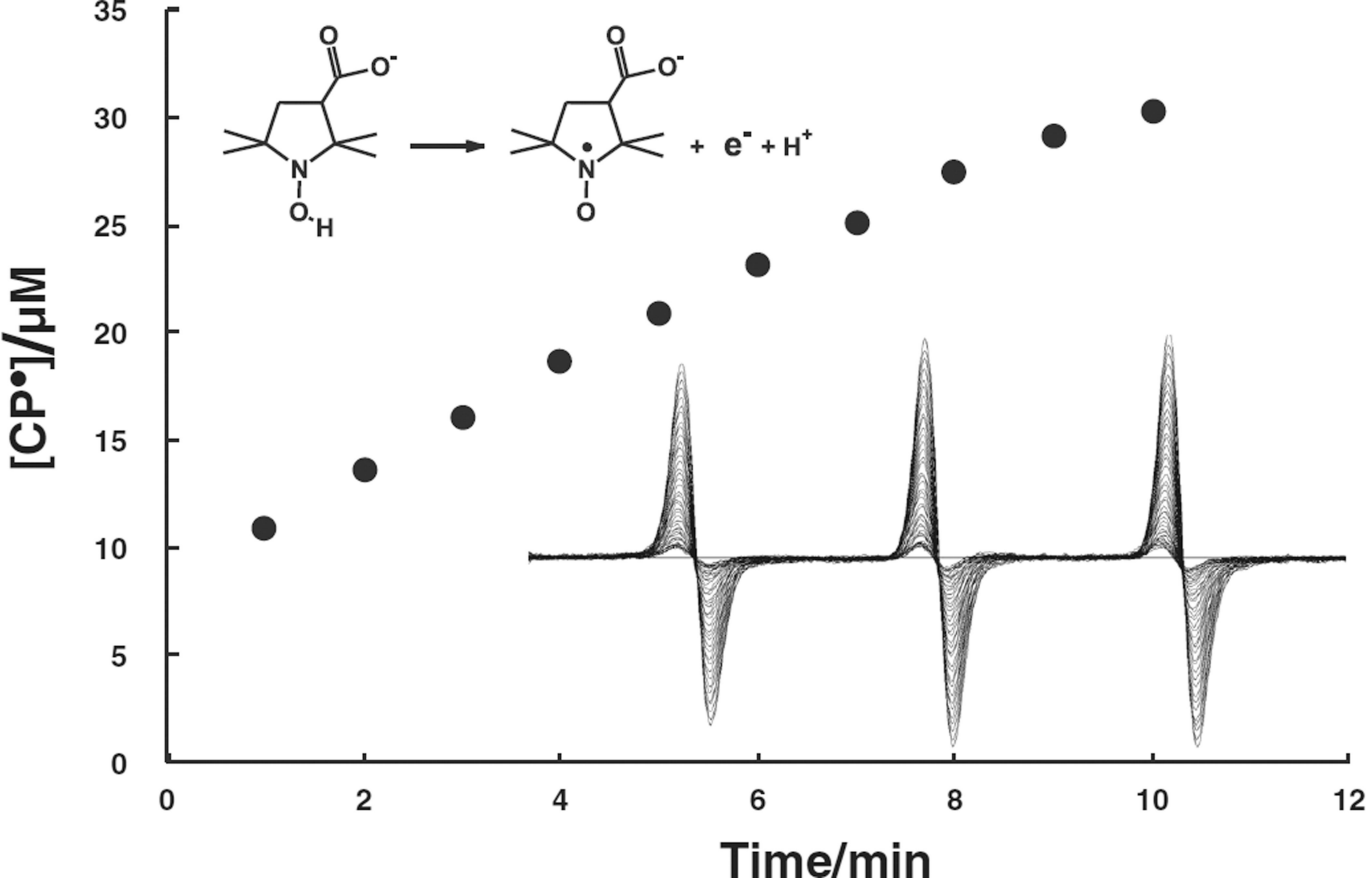
CPH is oxidized to CP^•^ by inorganic RVM. CPH (200 µM) was included in an aqueous suspension of RVM (pH 7.2) and the formation of CP^•^ (a^N^ = 16.2 G [25]) was observed by EPR. The intensity of the spectrum of CP^•^ increased with time demonstrating ongoing one-electron oxidation of CPH in the aqueous suspension of RVM. In the absence of RVM only a very low background spectrum of CP^•^ ([CP^•^] < 0.1 µM) was observed that did not change significantly in this same time-frame. Inset: spectra collected over time varied only in intensity with no observable changes in lineshape consistent with the one-electron oxidation of CPH to CP^•^, with no other significant chemistry

When CPH is included in a near-neutral aqueous suspension of RVM it undergoes a time-dependent, one-electron oxidation to CP^•^, as observed by EPR, Fig. 3. That the signal increases with time indicates that oxidations are ongoing, rather than occurring in a very short burst.

CPH and the membrane non-penetrable probe CAT1-H was chosen to probe for in vivo oxidations initiated by RVM. However, the nitroxide radical associated with the one-electron oxidation of CAT1-H could not be observed in the EPR imaging experiments, even at very high doses of RVM (100× daily human dose equivalent) whereas CP^•^ was readily observed in the EPR imaging experiments. Thus, CPH was chosen as a reporter molecule for the in vivo imaging experiments. CPH is oxidized by RVM and as a five-membered ring nitroxide CP^•^ is much more resistant than six-membered ring nitroxides (*e.g*. tempo and tempol) to reduction back to the corresponding hydroxylamine by physiological reductants such as ascorbate [20]; CP^•^ is also more resistant to reduction than most 5-membered imidazole and di-aza nitroxides [23].

### Oxidation of CPH to CP^•^ by RVM in vivo

#### Digestive system (Imaging)

**Fig. 4:**
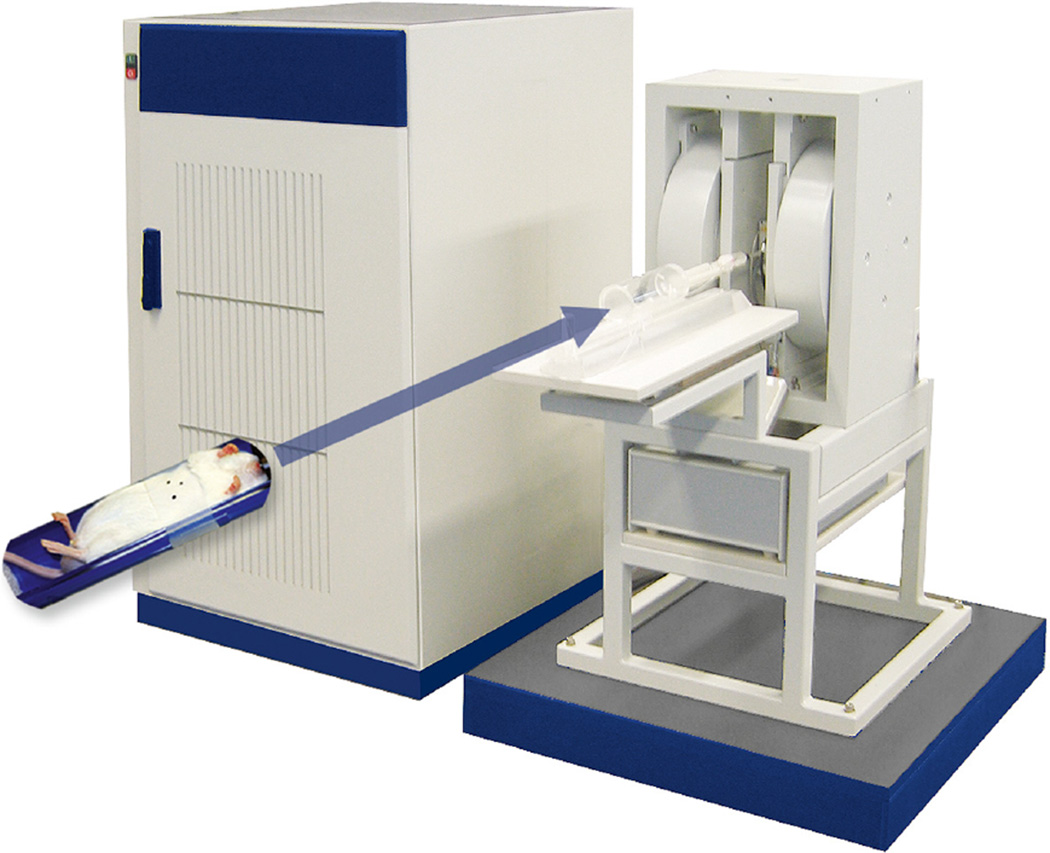
Configuration and area imaged by EPR to monitor *in vivo* formation of CP^•^. Anaesthetized (isoflurane 2.2 %) mice were positioned for imaging in a 36 mm cavity of L-Band EPR-Spectrometer ELEXSYS E540 equipped with an automatic matching control system. The area for imaging (30 × 30 mm) was chosen as presented (*blue circle* with *3 black dots*) to capture the digestive system. Acquisition of the spectra for CP^•^ in the digestion system was performed twice (2 × 10 min). EPR instrument settings were: center field, *g* = 2.01; field sweep, 60 G; microwave power, 40 mW; magnetic field modulation, 1 GHz; modulation amplitude, 3.0 G; conversion time, 20.24 ms; detector time constant, 40.96 ms; image field of view, 25 mm; acquired angles, 31; gradient, 24 G cm^−1^

**Fig. 5:**
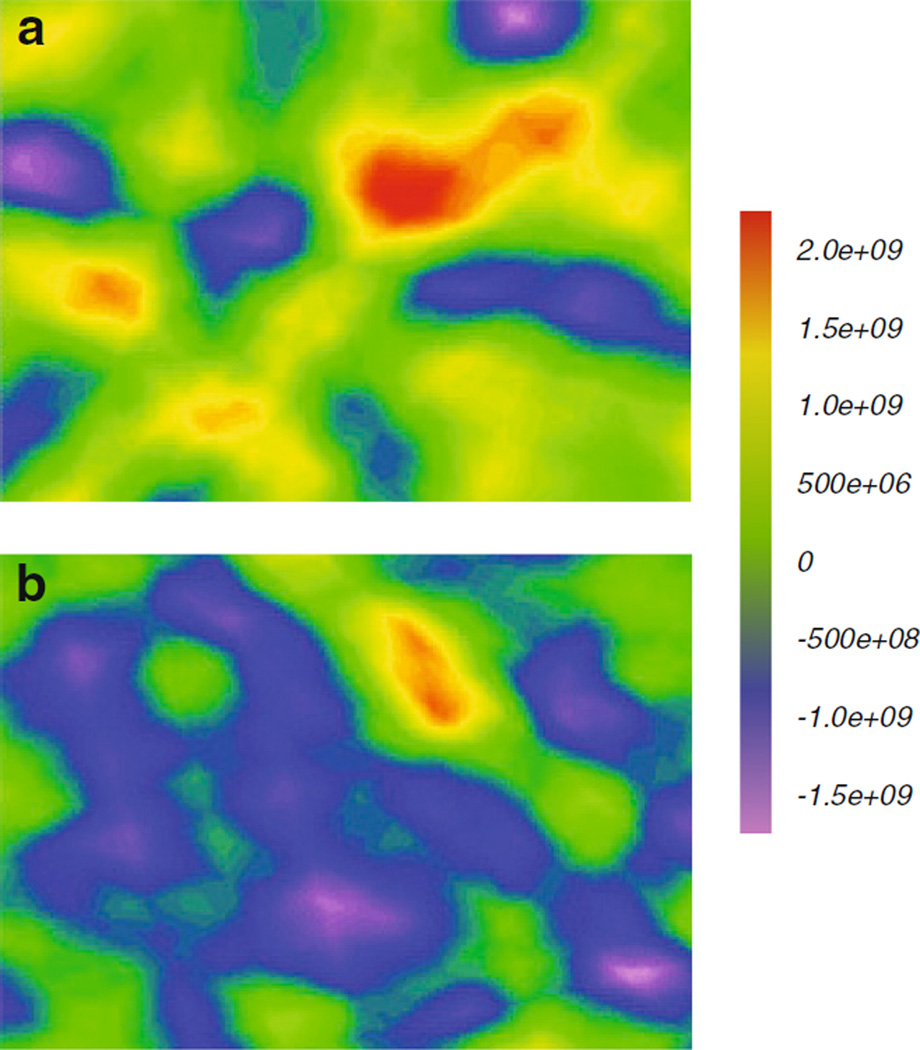
AAOS reduces oxidant flux from RVM as seen by *in vivo* EPR-imaging of free radical formation. RVM and CPH (1 mM) were administered per os and then mice were immediately imaged for formation of CP^•^; see Fig. 4. Co-administration of the inorganic formulation of RVM (**a**) and CPH shows much higher levels of CP^•^ than seen in the AAOS formulation of RVM (**b**) as seen by the prevalence of *red*/*yellow*/*green* areas in panel **a** compared the dominance of *violet*/*blue* areas of panel **b**, *i.e*. lower concentrations of CP^•^ in with the AAOS formulation. The scale on the *right* represents the intensity of EPR signal in A.U. From the data in Table 2, this range in concentration of CP^•^ radical is on the order of 0.1–235 µM

To determine if RVM initiates oxidations in vivo, we employed EPR imaging and the one-electron oxidation of CPH to CP^•^ as a reporter. Water suspensions of RVM formulations containing CPH were immediately administered into the digestive system of anesthetized mice by gavage. The mice were then immediately imaged by EPR, approximately 5 min after introduction of the materials, Fig. 4. We observed a higher concentration of CP^•^ in mice given the inorganic formulation of RVM than the formulation using AAOS, Fig. 5. Interesting was that EPR imaging showed that CP^•^ was present not only in the region of the digestive system but also in other parts of the image.

#### Systemic distribution of CP^•^ as seen by ex vivo analysis

Because EPR imaging indicated the presence of CP^•^ throughout the region being imaged, we examined the distribution of CP^•^ in the mouse after administration of RVM/CPH. Fifteen minutes after the introduction of the RVM formulations into the stomach of the mice, fluid and organ materials were harvested for analysis of CP^•^. The analysis showed wide distribution of CP^•^, Table 2 and Fig. 5. Blood had the highest level of CP^•^; as expected CP^•^ appeared in bladder fluid as well as fluids from other organs. The inorganic formulation of RVM resulted in nearly twice the level of CP^•^ as the AAOS formulation. When CPH was administered in the absence of RVM, only very low levels of CP^•^ were observed in all samples, indicating that the RVM formulations were responsible for the vast majority of CP^•^ that appeared, *i.e*. RVM oxidized CPH to of CP^•^ in vivo. When CP^•^ (only) was administered as a control, a very high level was seen in stomach fluid and it distributed to other sites in a pattern similar to that observed by RVM and CPH.

Because administration of CPH alone as a control showed very little CP^•^ in all regions tested, the observation of CP^•^ throughout the mouse is consistent with RVM initiating oxidations in the digestive system. We conclude that the free radicals formed in the digestive tract by consumed minerals partitioned into the blood and thereby became distributed throughout entire body.

## Conclusions

We have clearly demonstrated the value of using the oxidation of CPH to CP^•^ as a reporter for one-electron oxidations in vitro and in vivo. Using the rates of oxygen consumption and loss of ascorbate radical coupled with the oxidation of CPH to CP^•^ we clearly show that RVM will catalyze the formation of oxidants. These oxidants initiate the one-electron oxidation of “bystander” species; here as a bystander, the oxidation of CPH to CP^•^ is followed by EPR. In vivo, CP^•^ formed by the oxidation of CPH in the stomach and intestine spreads throughout the body, as demonstrated by the wide distribution of CP^•^.

We show that RVM catalyzes oxidations; the rate of these oxidations can be modulated by the materials used in the formulation of RVM. When AAOS is used in the formulation, as apposed to inorganic forms of minerals, the rate of oxidation in aqueous suspensions of RVM in vitro is decreased by at least 50 % as determined by the rates of oxygen uptake, loss of ascorbate radical, and formation of CP^•^. Real-time EPR imaging also demonstrates that AAOS blunts oxidations induced by multivitamin/multimineral supplements in vivo.

These results demonstrate that typical ingredients of multivitamin/multimineral supplements result in the oxidation of materials within the formulation as demonstrated by the oxidation of ascorbate, but also bystander materials can be oxidized as demonstrated by the oxidation of CPH to CP^•^. We clearly show both in vitro and in vivo that the nature of the coordinating ligands of the redox-active minerals is an important consideration. We have demonstrated both in vitro and in vivo that AAOS greatly reduces the rate of oxidations initiated by the minerals in typical multivitamin/multimineral supplements.

## Figures and Tables

**Table 1:** Formulations of the two different RVM supplements

Ingredient	Amount per serving	% Daily value^a^
Common ingredients		
Vitamin A (as beta carotene)	5000 IU	100 %
Vitamin C (as ascorbic acid)	60 mg	100 %
Vitamin D (as cholecalciferol)	400 IU	100 %
Vitamin E (as d-alpha tocopheryl succinate)	30 IU	100 %
Vitamin K (as phytonadione)	80 µg	100 %
Thiamin (as thiamin HCl)	1.5 mg	100 %
Riboflavin	1.7 mg	100 %
Niacin (as niacinamide)	20 mg	100 %
Vitamin B6 (as pyridoxine HCl)	2 mg	100 %
Folate (as folic acid)	400 µg	100 %
Vitamin B12 (as cyanocobalamin)	6 µg	100 %
Biotin (as d-biotin)	300 µg	100 %
Pantothenic Acid (as calcium pantothenate)	10 mg	100 %
Calcium (as calcium carbonate)	100 mg	10 %
Magnesium (as magnesium oxide)	40 mg	10 %
Iodine (as potassium iodide)	150 µg	100 %
Microcrystalline cellulose	121 mg	na^b^
Croscaramellose sodium	12 mg	na
Silicone dioxide	12 mg	na
Magnesium stearate	4 mg	na
Carnauba wax	0.14 mg	na
RVM #1- Inorganic		
Iron (as iron sulfate)	18 mg	100 %
Zinc (as zinc sulfate)	15 mg	100 %
Selenium (as L-selenomethionine)	70 µg	100 %
Copper (as copper sulfate)	2 mg	100 %
Manganese (as manganese sulfate)	2 mg	100 %
Chromium (as chromium chloride)	120 µg	100 %
Molybdenum (as sodium molybdate)	75 µg	100 %
RVM #2- AAOS		
Iron (as iron AAOS)	18 mg	100 %
Zinc (as zinc AAOS)	15 mg	100 %
Selenium (as selenium AAOS)	70 µg	100 %
Copper (as copper AAOS)	2 mg	100 %
Manganese (as manganese AAOS)	2 mg	100 %
Chromium (as chromium AAOS)	120 µg	100 %
Molybdenum (as molybdenum AAOS)	75 µg	100 %

a % Daily Values (%DV) are based on Daily Value recommendations for key nutrients based on 2000 cal daily diet

b na is not applicable

**Table 2:** Tissue distribution of CP^•^ after administration of RVM and CPH

Treatment	Stomach [CP^•^]/µM^a^	Intestine	Blood	Bladder
CPH^b^,^c^	0.93 ± 0.13	1.3 ± 0.1	2.0 ± 0.4	0.44 ± 0.02
CP^•^ b,c	279 ± 11	11 ± 1	284 ± 15	21 ± 2
CPH + RVM-Inorganic^d^	0.52 ± 0.10	0.47 ± 0.02	235 ± 23	26 ± 3
CPH + RVM-AAOS^d^	0.27 ± 0.06^e^	0.25 ± 0.01^e^	138 ± 5^e^	18 ± 1^e^

a Concentration of CP^•^ observed in fluids harvested 15 min after treatment. Data are average ± standard error of mean

b 1.0 mM of CPH or CP^•^ (200 µL per 20 g BW); see Methods

c *n* = 3 for each determination

d *n* = 5 for each determination

e *p* < 0.05 when compared to the corresponding results from CPH + RVM-Inorganic, above

## Abbreviations

AAOS: Amino acid/oligosaccharide
CAT1H: 1-Hydroxy-2,2,6,6-tetramethylpiperidin-4-yl-trimethylammonium chloride•HCl
CPH: 1-Hydroxy-3-carboxy- 2,2,5,5-tetramethylpyrrolidine
CP^•^: 3-Carboxy-2,2,5,5-tetramethyl-1-pyrrolidinyloxy
DETC: Diethyldithiocarbamate
DFO: Deferoxamine
EPR: Electron paramagnetic resonance
OSL: Oxygen-Sensitive Label, tetrathiatriarylmethyl radical
RVM: Research vitamin mineral formulation
